# N6-methyladenosine Modification of Hepatitis B Virus RNA in the Coding Region of *HBx*

**DOI:** 10.3390/ijms24032265

**Published:** 2023-01-23

**Authors:** Takayuki Murata, Satoko Iwahori, Yusuke Okuno, Hironori Nishitsuji, Yusuke Yanagi, Koichi Watashi, Takaji Wakita, Hiroshi Kimura, Kunitada Shimotohno

**Affiliations:** 1 Department of Virology and Parasitology, Fujita Health University School of Medicine, Toyoake 470-1192, Japan; 2 Department of Virology, Nagoya University Graduate School of Medicine, Nagoya 466-8550, Japan; 3 Department of Virology, Nagoya City University Graduate School of Medical Sciences, Nagoya 467-8601, Japan; 4 Research Center for Hepatitis and Immunology, National Center for Global Health and Medicine, Ichikawa 272-8516, Japan; 5 Department of Virology II, National Institute of Infectious Diseases, Tokyo 162-8640, Japan; 6 Research Center for Drug and Vaccine Development, National Institute of Infectious Diseases, Tokyo 162-8640, Japan

**Keywords:** HBV, RNA, m6A modification, primary hepatocyte

## Abstract

N6-methyladenosine (m^6^A) is a post-transcriptional modification of RNA involved in transcript transport, degradation, translation, and splicing. We found that HBV RNA is modified by m^6^A predominantly in the coding region of *HBx*. The mutagenesis of methylation sites reduced the HBV mRNA and HBs protein levels. The suppression of m^6^A by an inhibitor or knockdown in primary hepatocytes decreased the viral RNA and HBs protein levels in the medium. These results suggest that the m^6^A modification of HBV RNA is needed for the efficient replication of HBV in hepatocytes.

## 1. Introduction

Hepatitis B virus (HBV) is a hepato-tropic enveloped virus with a DNA genome of about 3.5 kb that belongs to the family hepadnaviridae, genus orthohepadnavirus. The virus is transmitted via blood and can be sexually acquired. It is estimated that about two billion people are infected with HBV and 300 million are persistently infected worldwide [1]. HBV is prevalent mostly in developing countries, but it is also present in developed countries. Symptoms of hepatitis caused by HBV infection include fever, nausea, hypochondrial pain, and jaundice. After chronic hepatitis, some patients develop liver cirrhosis or liver cancer [2,3]. An inactivated HBV vaccine is available worldwide, and immunoglobulin preparations can also be used. For treatment, interferons and reverse transcriptase inhibitors are used to suppress HBV. However, in many cases, these drugs do not completely eliminate HBV. Therefore, novel, effective antivirals are needed. However, the development of new antivirals is hampered by the complex lifecycle of HBV and an incomplete understanding of the mechanisms underlying its replication and persistence [4,5].

After transcription, RNAs, including mRNAs, are subject to chemical modifications. One of the most common RNA modifications is N^6^-methyladenosine (m^6^A), i.e., the methylation of adenosine residues in RNA. The m^6^A modification has many physiological functions, including RNA processing, nuclear export, decay, and translation [6,7]. The genomes and mRNAs of many RNA viruses have the m^6^A modification [8,9,10,11]. For example, human immunodeficiency virus type 1 RNA is modified by m^6^A in the 3’-region, which enhances viral gene expression [12,13]. The RNAs of flaviviruses, such as hepatitis C virus and Zika virus, as well as influenza virus and Kaposi’s sarcoma-associated herpesvirus, also have the m^6^A modification [14,15,16,17].

Because the m^6^A modification of HBV RNA had not been reported when we started this work, we identified methylation site(s) in HBV RNA. However, during our work, a paper on m^6^A modification of HBV RNA was published by another group [18]. Nevertheless, we continued to independently analyze the methylation sites and physiological role of methylation of HBV, because the methylation site in the report (adenosine residue in the stem loop of epsilon) did not coincide with the methylated adenosines that we identified. We identified four major m^6^A adenosines in the coding region of *HBx*. Inhibition of m^6^A modification by an inhibitor, knockdown of the methylation enzyme, and mutagenesis of the m^6^A sites reduced viral RNA and protein levels in primary human hepatocytes (PHHs). Therefore, the m^6^A modification in HBV RNA promotes HBV replication in primary hepatocytes.

## 2. Results

### 2.1. m^6^A Modification of HBV RNA

**Figure 1:**
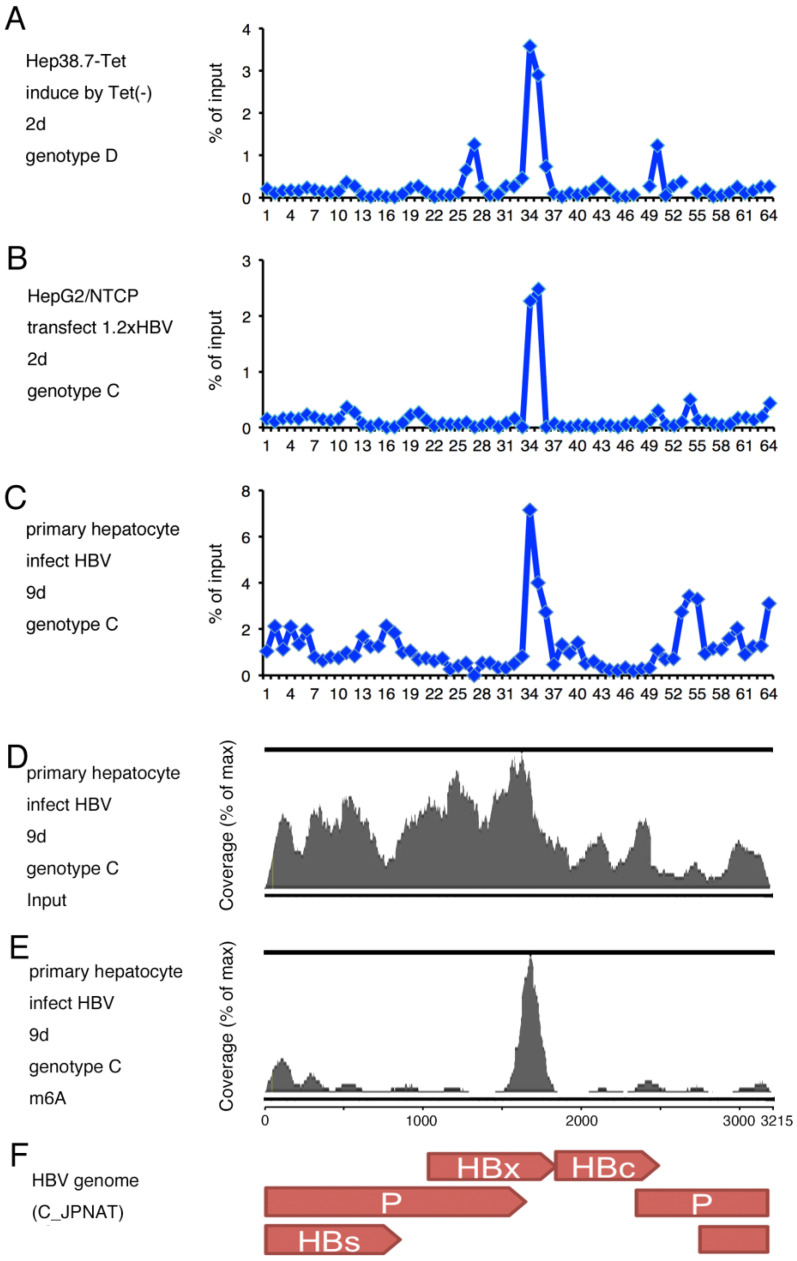
**Modification of HBV RNA by m^6^A**. (**A**) Transcription of HBV RNA was induced by removing tetracycline from the culture medium of Hep38.7-Tet cells for 2 days. (**B**) HepG2/NTCP cells were transfected with pUC1.2xHBV and incubated for 2 days. (**C**–**E**) Primary human hepatocytes (PHHs) were infected with HBV with 4% PEG and incubated for 9 days. (**A**–**C**) RNA was isolated and subjected to MeRIP analysis followed by qRT-PCR. We prepared 64 sets of oligonucleotide primers that covered the whole HBV pregenome RNA. The precipitated RNA level was plotted after normalization to the input level as percentage of input. (**D**,**E**) RNA was isolated and subjected to MeRIP analysis, which was followed by RNA-seq. Reads from input (**D**) and MeRIP-precipitated (**E**) samples were mapped to the reference HBV strain (C_JPNAT) and shown as coverage (%). (**F**) Schematic of HBV genes in the reference strain.

To determine if HBV RNA is modified by m^6^A, we performed a methylated RNA immunoprecipitation (MeRIP) analysis using Hep38.7-Tet cells (Figure 1A). The whole genome sequence of HBV genotype D (AD38) is stably integrated into the cell line, whose expression is tightly regulated in the presence of tetracycline and induced by its removal. MeRIP-qRT-PCR was carried out by using RNA sample collected at 2 days after removal of tetracycline. Sixty-four pairs of primers were used for the qRT-PCR so that the fragments evenly cover the HBV whole genome of AD38. The percentage of precipitated RNA was plotted after normalization to the input level. A major peak was detected with primer sets #34 and #35, which targeted the C-terminus of the HBx coding region (Figure 1A). In addition, we observed several minor peaks, too. However, neither of the peaks correspond to the m^6^A site reported by the other group [18]. 

To examine the methylation sites under other conditions, HepG2/NTCP cells were transfected with the 1.2xHBV plasmid vector, and MeRIP was carried out after 2 days (Figure 1B). The 1.2xHBV plasmid contains 1.2 copies of the wild-type HBV genome (C_JPNAT, genotype C). Because of the overlapping promoter region for pregenome transcription, HBV RNA is produced upon transfection. MeRIP assay indicated that the methylation peaked at #34 and #35 under this condition as well.

We analyzed the m^6^A level in primary hepatocytes after HBV infection (Figure 1C) because established cell lines, e.g., HepG2 cells, and artificial induction of HBV RNA (Tet regulation and transfection of 1.2xHBV plasmid) might show unnatural methylation. We detected a major peak in the same region in PHH sample (#34 and #35). Therefore, HBV RNA is m^6^A-modified predominantly at the C-terminus of HBx, irrespective of cell type and condition.

To validate the results, RNA-seq analyses of MeRIP product and input RNA were conducted using the sample of PHHs infected with HBV (Figure 1D,E). The highest m^6^A peak was in the HBx-coding region (1611–1764 of AB246345.1), which corresponds to the peaks at #34 and #35 (Figure 1E). Figure 1F shows a schematic diagram of HBV genes. 

### 2.2. Identification of m^6^A Modified Residues in HBV RNA

**Figure 2:**
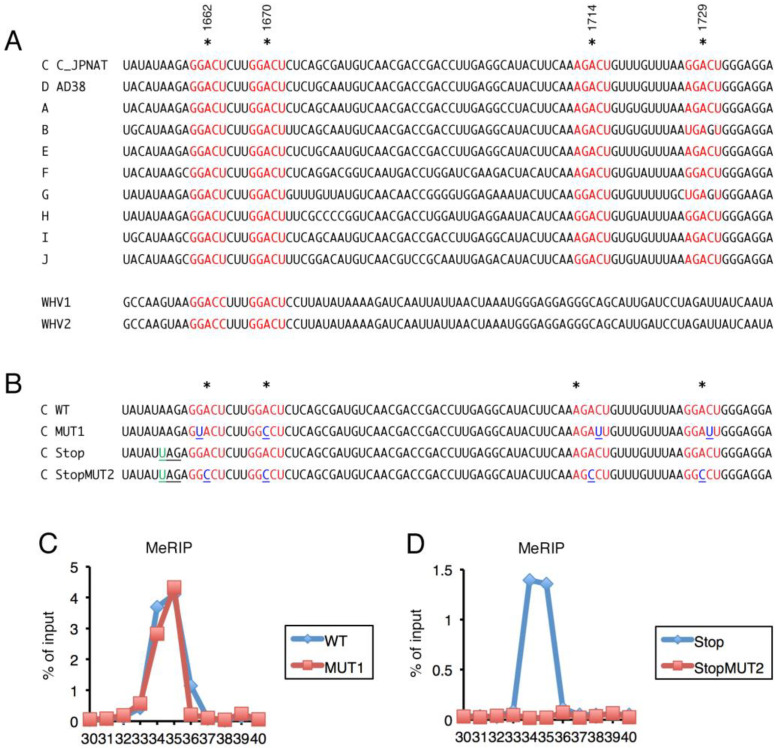
**Identification of m^6^A sites in HBV RNA.** (**A**) Sequence alignment of the region for predominant m^6^A modifications (#34 and #35) in HBV (C_JPNAT (genotype C), AD38 (genotype D), and other genotypes) and Woodchuck hepatitis viruses (WHV1 and WHV2). Asterisks denote possible m^6^A sites. Red letters indicate conserved consensus motifs. (**B**) Mutagenesis of possible m^6^A sites. Mutated nucleotides were indicated by underlines. The AAG sequence upstream of A1662 was modified to UAG to change the codon to Stop. (**C**,**D**) HepG2/NTCP cells were transfected with pUC1.2xHBV with the indicated mutations, incubated for 2 days, and subjected to MeRIP analysis, which was followed by qRT-PCR.

The RNA sequence of the major m^6^A peak of HBV (Figure 1, #34 and #35) is shown in Figure 2A. The 103 bp sequence has four possible methylation sites in the m^6^A consensus motif (A/G/U-G/A-A-C-A/C/U; the underlined adenosine residue is methylated): A1662, A1670, A1714, and A1729 of HBV C_JPNAT (AB246345). Three of the four possible m^6^A consensus motifs (A1662, A1670, and A1714) are conserved not only in genotypes C and D (used in Figure 1), but also in other genotypes. The A1729 consensus motif was imperfectly retained (black-colored G at 1730 in the Figure 2A). Interestingly, A1662 and A1670, and the surrounding consensus sequences, are preserved in woodchuck hepatitis viruses (Figure 2A), suggesting the importance throughout the orthohepadnavirus group.

We next introduced mutations into the consensus motifs of possible m^6^A sites. Because the 103-bp sequence (#34 and #35) is in the coding region of *HBx*, we firstly disrupted the consensus sequence while preserving the codons of HBx (Figure 2B, MUT1). Note that only one possible methylation acceptor adenosine (A1670) could be directly mutated, while A1662, A1714, and A1729 were not mutated and their surrounding nucleotides were mutated (Figure 2B, MUT1). Transfection of the 1.2xHBV plasmid with the MUT1 sequence resulted in a similar m^6^A modification level as WT 1.2xHBV (Figure 2C). Therefore, we next mutated the four adenosine residues to cytosines. However, these mutations altered some amino acids of HBx. Because HBx reportedly affected the m^6^A modification [19], we evaluated the m6A level in the absence of HBx by inserting a stop codon just before A1662 both in the control and A/C mutant (Figure 2B, Stop and StopMUT2, respectively). In other words, both Stop and StopMUT2 express truncated HBx protein of the same size. Direct mutation of the four adenosines (StopMUT2) markedly reduced the m^6^A level (Figure 2D, rouge), indicating that the four residues are acceptors of m^6^A. 

We attempted to determine which of the four adenosines is modified by m^6^A. However, neither of the single mutations of the adenines reduced the modification level (not shown), suggesting that the m^6^A modifications of the four adenosines are redundant.

### 2.3. Mutation of m6A Sites Decreased HBV RNA and HBs Levels

**Figure 3:**
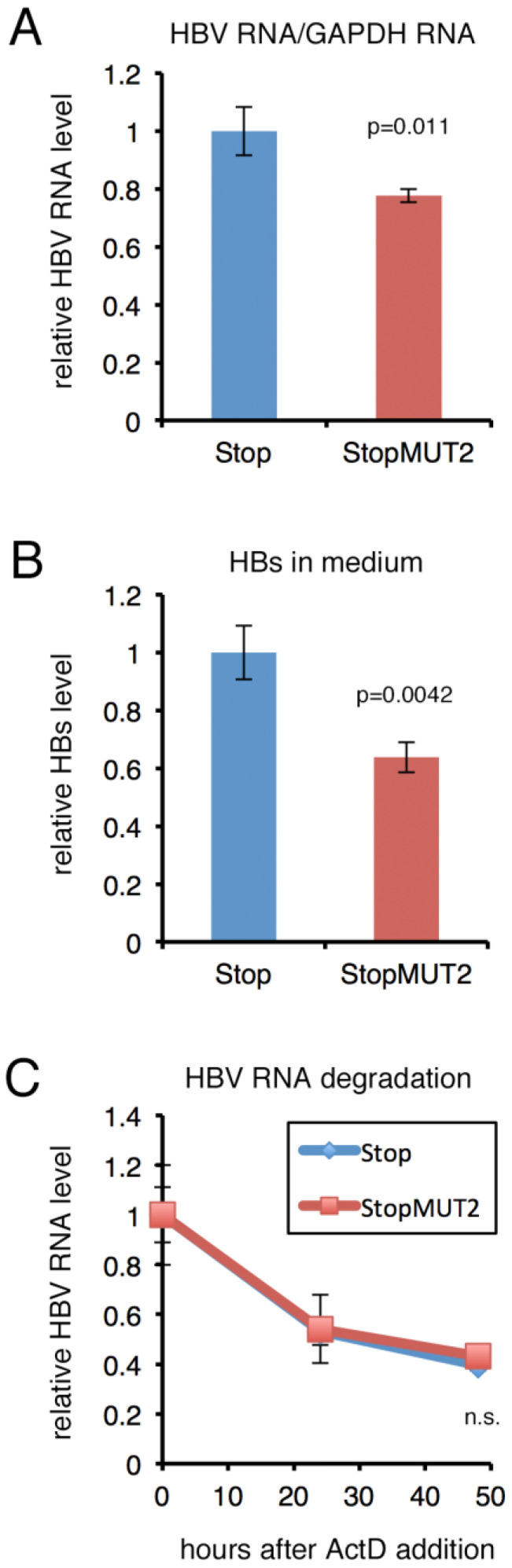
**Effect of m^6^A-null mutation in HBV RNA.** (**A**) HepG2/NTCP cells were transfected with pUC1.2xHBV (Stop) or pUC1.2xHBV with m^6^A-null mutation (StopMUT2). RNA was harvested after 2 days and qRT-PCR analysis was performed to quantitate RNA levels. (**B**) HepG2/NTCP cells were transfected with the same plasmids. Culture media were collected after 2 days for HBs ELISA. (**C**) HepG2/NTCP cells were transfected with the same plasmids. The next day, actinomycin D (5 μg/mL) was added, which was followed by incubation for 24 or 48 h. RNA was harvested, which was followed by qRT-PCR analysis. Relative HBV RNA levels are normalized to the levels at 0 h and plotted. Means ± standard deviation (SD) of three independent biological replicates are shown. Student’s *t*-test was used for the analysis.

To analyze the effects of the four mutations at m^6^A sites, HepG2/NTCP cells were transfected with the 1.2xHBV plasmid (Stop or StopMUT2). After 2 days, quantitative reverse transcription-polymerase chain reaction (qRT-PCR) (Figure 3A) and enzyme-linked immunosorbent assay (ELISA) (Figure 3B) were carried out. HBV RNA levels were decreased slightly but significantly (Figure 3A). The HBs protein level in medium was decreased by 36% (Figure 3B). To examine if mutations at the m^6^A sites affected HBV RNA degradation, de novo RNA synthesis was halted by actinomycin D, which was followed by incubation for 24 and 48 hours (Figure 3C). The degradation rate was similar between Stop and StopMUT2, indicating that the reduced HBV RNA level (Figure 3A) cannot be accounted for by a change in degradation rate.

### 2.4. Inhibition of m^6^A Modification Resulted in Lower HBV RNA and HBs Levels in PHH

**Figure 4:**
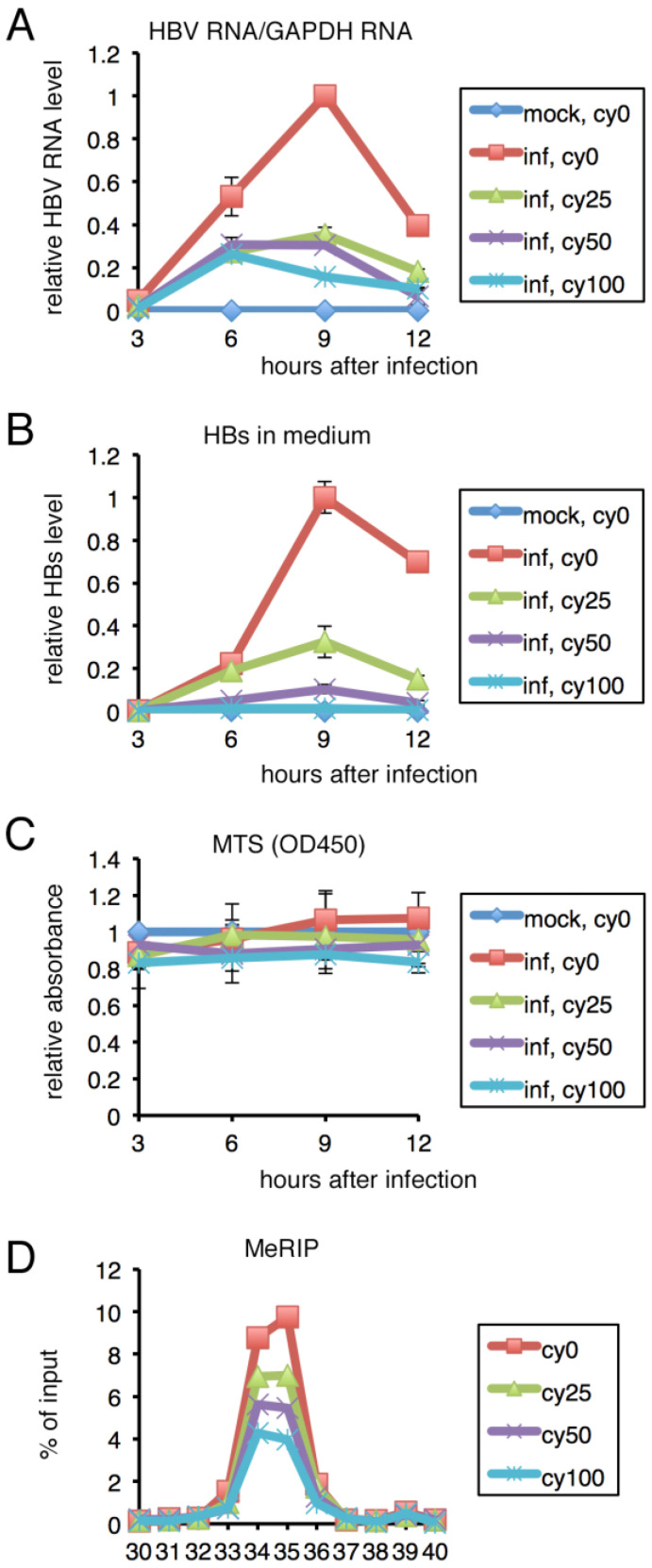
**Effect of cycloleucine in PHHs.** (**A**–**D**) PHHs were pretreated with the indicated concentrations of cycloleucine (mM) for 1 h and infected with HBV in the presence of 4% PEG and cycloleucine. RNA and culture medium were harvested for qRT-PCR (**A**) and HBs ELISA (**B**), respectively. MTS assay was conducted to examine cell viability (**C**). (**A**–**C**) Means ± standard deviation (SD) of three independent biological replicates are shown. (**D**) RNA was isolated from the cells and subjected to MeRIP analysis, which was followed by qRT-PCR.

We next evaluated the effect of the m^6^A methylation inhibitor, cycloleucine, on HBV in PHHs. Levels of HBV RNA and HBs production were monitored by qRT-PCR and ELISA (Figure 4). Cycloleucine decreased the HBV RNA level in cells (Figure 4A) and HBs level in medium (Figure 4B) in a dose-dependent manner, whereas 3-[4,5-dimethylthiazol-2-yl]-5-[3-carboxymethoxyphenyl]-2-[4-sulfophenyl]-2H-tetrazolium (MTS) assay demonstrated that cell viability was unaffected (Figure 4C). MeRIP analysis confirmed that the m^6^A modification was suppressed by cycloleucine (Figure 4D), although the effect of cycloleucine appeared not very strong, as the methylation was reduced by only about 50% even at the highest concentration. 

### 2.5. Knockdown of METTL3 Decreased HBV RNA and HBs Levels in PHH

**Figure 5:**
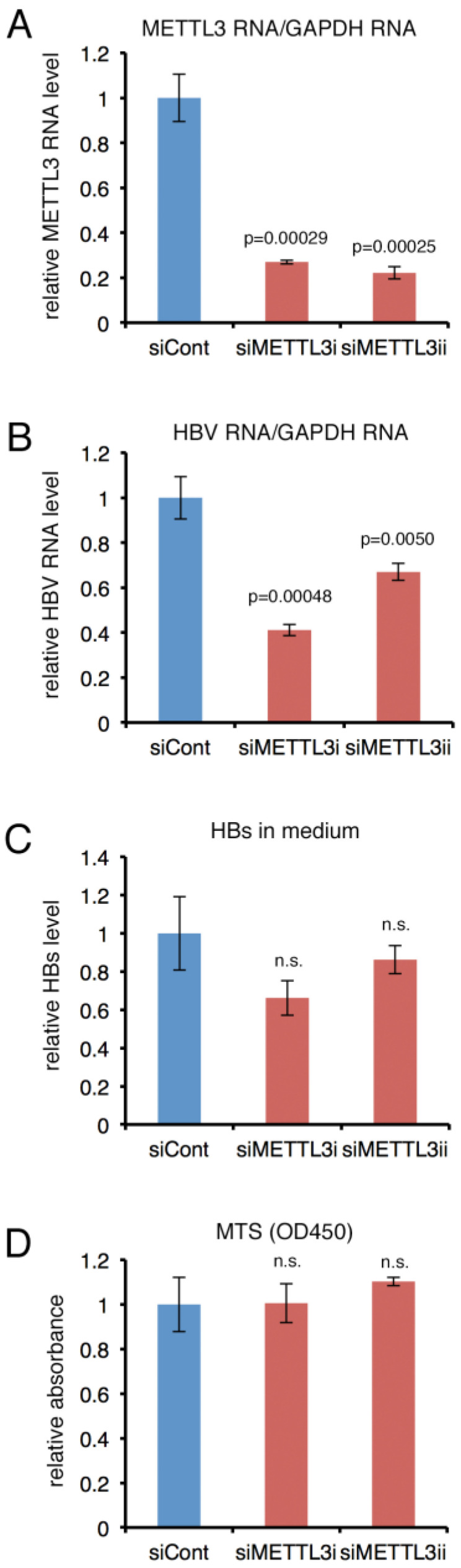
**Effect of knockdown of *METTL3* in PHH.** (**A**–**D**) PHHs were transfected with a control siRNA (siCont) or siRNA for METTL3 (siMETTL3i, ii). The next day, cells were infected with HBV in the presence of 4% PEG. At 3 days after infection, siRNA was again transfected, and RNA and culture medium were harvested at day 7 for qRT-PCR (**A**,**B**) and HBs ELISA (**C**), respectively. MTS assay was conducted to examine cell viability (**D**). Means ± standard deviation (SD) of three independent biological replicates are shown. Student’s *t*-test was used for the analysis.

We examined whether knockdown of the m^6^A methylation enzyme METTL3 by small interfering RNA (siRNA) transfection influenced HBV gene expression in PHHs. To this end, two independent siRNAs were designated and synthesized (Figure 5, siMETTL3i and siMETTL3ii). Compared to the control, both siMETTL3i and siMETTL3ii successfully reduced the METTL3 level to 24 and 22%, respectively (Figure 5A). Under this condition, the knockdown of METTL3 decreased the HBV RNA level to 41% and 67% (Figure 5B). The HBs protein level in medium was lower in the knockdown sample, albeit not significantly (Figure 5C). An MTS assay confirmed that cell viability was unaffected by the transfection of either of the siRNAs (Figure 5D). 

### 2.6. Expression of HBx Was Not Involved in m^6^A Modification

**Figure 6:**
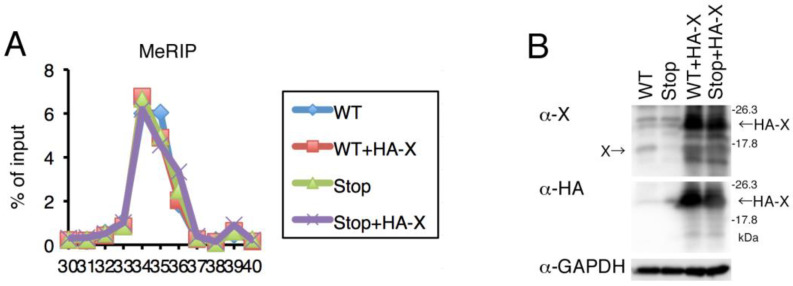
**Effect of HBx on HBV RNA methylation.** (**A**) HepG2/NTCP cells were co-transfected with pUC1.2xHBV (WT) or its HBx-Stop mutation (Stop) together with HA-tagged HBx expression vector or its empty control vector. After 2 days, RNA was isolated and subjected to MeRIP analysis, which was followed by qRT-PCR. The level of precipitated RNA was plotted according to input level. (**B**) Protein level of HBx. Western blotting was carried out using anti-HBx, -HA, and -GAPDH antibodies.

Since it was reported that HBx was crucial in m^6^A modification of viral RNA [19], we tested whether methylation was affected by disruption of the *HBx* gene or exogenous overexpression of the gene product. The WT or Stop strain of 1.2xHBV shown in Figure 2 was co-transfected with an HA-tagged HBx expression vector or its empty vector. A stop codon was artificially introduced into the *HBx* gene in the Stop mutant, but the methylated adenosine residues (A1662, A1670, A1714, and A1729) were intact. Two days after transfection, RNA was harvested and subjected to MeRIP assay (Figure 6). Loss of HBx protein by point mutation (Stop) had little or no effect on HBV RNA methylation when compared to WT (Figure 6A). Western blotting confirmed that the X protein was present in the WT sample, while the band was very weak in the Stop sample (Figure 6B, anti-X). The m^6^A level of HBV RNA was not increased nor decreased at all by the exogenous supply of the protein (+HA-X) to either WT or Stop (Figure 6A). The production of HA-tagged HBx protein was confirmed by Western blotting using anti-HA antibody (Figure 6B).

## 3. Discussion

Here, we first carried out MeRIP assays under several conditions. MeRIP assays identified several minor differences between genotypes and cell types, but the major peak was in the C-terminal coding region of *HBx* (Figure 1). It was reported that HBV RNA was modified by m^6^A at an adenosine residue in the epsilon stem loop (A1907) [18]. The A1907 in the previous report corresponds to A1905 of our reference strain (C_JPNAT), and this sequence is present in #39 of our primer sets (Figure 1). No obvious peak was detected at/around #39 in our experiments (Figure 1), except that a very faint increase might be present at #39 in PHHs infected with HBV when detected by MeRIP-qRT-PCR (Figure 1C). We are confident in our results because the substitution of four adenosines (A1662, A1670, A1714, and A1729) attenuated the peak (Figure 2). An online m^6^A prediction algorithm [20] suggests that the four adenosines are methylated with high confidence (A1662 and A1670) and very high confidence (A1714 and A1729) in the liver, whereas the adenosine residue, reportedly an m^6^A target [18], was not predicted. The sequences surrounding the four adenosines are highly conserved (Figure 2). Moreover, the m^6^A modification most frequently occurs around the stop codon and in the 3’-UTR [21], and the four adenosines are located in the 3’-UTRs of *HBc*, *P*, and *HBs* mRNAs, and near the stop codon in *HBx* mRNA. We compared the difference in more details between the previous report [18] and us. We used the same poly(A) RNA isolation kit with them, and the Hep38.7-Tet cell line we used in Figure 1A appeared to be the subclone of HepAD38, which was the cell line they used in the previous report [18]. One obvious difference is in the MeRIP process; we used a commercial kit from Merck, while they seemed to establish MeRIP experiments by themselves by using a purchased antibody and Dynabeads. It is unclear why our m^6^A sites did not coincide with the nucleotides reported previously, but the discrepancy might be due to the conditions and/or methods used.

Identification of the methylated adenosine residues was not simple in our manuscript because there were four highly possible adenosines accumulated in the peak and also because they are in the open reading frame of HBx. Because a single mutation of the possible adenosines had little or no effect on m^6^A methylation (Figure 2), we speculate that the four adenosines are redundantly methylated.

Using the m^6^A null mutant, we analyzed the physiological role of methylation in HepG2 cells (Figure 3). The HBV RNA level in cells and HBs level in medium were decreased by the null mutation. The pharmacological inhibition of methylation reduced the HBV RNA level in cells, and the HBs protein level in medium, in the context of HBV infection of PHHs (Figure 4). Knockdown of the responsible methylation enzyme, METTL3, also reduced HBV RNA and HBs expression in PHHs (Figure 5). These data contradict a previous report in which METTL3/14 knockdown stabilized HBV RNA and thereby reinforced viral protein expression in HepG2 cells [18]. However, for the knockdown experiment, we realize a difference; they used HepAD38, while we used PHHs. So, the difference in physiological roles of the methylation may be explained by cell type difference.

Furthermore, we could not reproduce the finding of the same group that HBx regulates m^6^A modifications (Figure 6) [19]. However, these contradictory results can be attributed to the cell types used. This study presented data for PHHs because they are similar to liver cells in vivo.

We admit this study had technical limitations, especially with respect to the analysis of the physiological role of HBV RNA methylation. The pharmacological inhibition and knockdown of METTL3 disrupts many m^6^A target RNAs besides HBV RNA, which may indirectly affect the HBV lifecycle. Mutagenesis of the target adenosine residues in HBV can exert direct effects (Figure 3), which could still be influenced by artifacts arising from unequal transfection efficiency or unexpected effect of the mutation; i.e., the mutated adenosines might somehow be needed for the optimal transcription of HBV RNA as well as for m^6^A methylation.

The m^6^A modification and G-quadruplexes of viral RNAs may occur simultaneously [22]. G-quadruplex formation may affect the m^6^A modification, and the G-quadruplex structure may be influenced by m^6^A. According to a prior report, the m^6^A sequence (A1907 in the epsilon stem loop) [18] may form a G-quadruplex because the sequence around the site has a possible G-quadruplex motif: G_n_-L_x_-G_n_-L_x_-G_n_-L_x_-G_n_, where n ≥ 2, and x = 1–20. The sequences around our m^6^A sites were compatible with the formula, so there may be a relationship between G-quadruplex formation and m^6^A.

Genes related to the m^6^A modification, such as METTL3 and YTHDF1, are upregulated in hepatocellular carcinoma (HCC) and associated with a worse prognosis [23,24]. The m^6^A modification by MELLT3 promotes liver cancer progression [25]. In HBV-associated HCC, the expression of other components, including RBM15 and HNRNPA2B1, was correlated with overall survival [26]. In addition, because an inhibition of m^6^A modification in PHH decreased HBV infection (Figure 4 and Figure 5), m^6^A modification may be a novel target for drug treatments of HCC, especially HBV-associated HCC. Further studies are needed to develop novel antiviral/anticancer drugs targeting the m^6^A modification.

## 4. Materials and Methods

### 4.1. Cell Culture and Reagents

PHH cells, culture media, and HBV obtained from the sera of chimeric mice were purchased from PhoenixBio. HepG2/NTCP cells [27] were cultured in DMEM (Life Technologies, Carlsbad, CA, USA) supplemented with 10% FBS, penicillin, and streptomycin. Hep38.7-Tet cells [28], a subclone of HepAD38 [29], were cultured in DMEM/F12 (ThermoFisher Scientific, Waltham, MA, USA) supplemented with 10% FBS, insulin (FUJIFILM Wako, Tokyo, Japan), penicillin, streptomycin (Sigma-Aldrich), and tetracycline (Sigma-Aldrich, St. Louis, MO, USA). Cycloleucine and actinomycin D were obtained from Sigma-Aldrich and Nacalai Tesque (Kyoto, Japan), respectively. Anti-HBx, -HA, and -GAPDH antibodies were purchased from Abcam (Cambridge, UK), Sigma-Aldrich, and Cell Signaling Technology (Danvers, MA, USA), respectively.

### 4.2. Plasmids

We used pUC1.2xHBV [30], in which a 1.2-fold HBV genome (isolate C_JPNAT, genotype C, accession #AB246345) was cloned. Site-directed mutagenesis was carried out by a PCR-based method. An HA-tagged HBx expression vector was prepared by inserting an HA and *HBx* gene open-reading frame into pcDNA (ThermoFisher Scientific). 

### 4.3. qRT-PCR

RNA was isolated using the RNeasy Mini Kit (Qiagen, Venlo, Netherlands), and qRT-PCR was performed using the One-Step SYBR PrimeScript RT-PCR Kit II (TAKARA BIO, Kusatsu, Japan) and Real-Time PCR System 7300 (ThermoFisher Scientific). To quantify HBV RNA, primers were designed for the preC/C region of HBV. The oligonucleotide primers used for the MeRIP-qRT-PCR assays will be disclosed upon request. Sixty-four pairs of primers were set to amplify fragments that were 50 bp long, covering the whole HBV genome (HBV C_JPNAT, accession #AB246345, genotype C or HBV AD38, transduced into Hep38.7-Tet, genotype D). 

### 4.4. MeRIP-qRT-PCR and MeRIP-RNAseq

MeRIP was carried out as described previously [31]. Cellular RNA was isolated using the RNeasy Mini Kit (Qiagen), which was followed by poly(A) RNA enrichment with the PolyA Spin mRNA Isolation Kit (New England Biolabs, Ipswich, MA, USA). After the fragmentation of poly(A) RNA, a portion (10%) of the RNA sample was retained to prepare the input control; the rest was subjected to immunoprecipitation using the Magna MeRIP m6A Kit (Merck Millipore, Burlington, MA, USA). qRT-PCR was performed using 64 sets of primers that covered the whole viral genome. For RNA-seq, sequencing libraries were prepared from both input RNA and MeRIP-purified RNA samples using the NEBNext Ultra II RNA Prep Kit for Illumina (New England Biolabs). The libraries were sequenced on a HiSeq X next-generation sequencer (Illumina, San Diego, CA, USA) with the 2 × 150 bp pair read option. The reads were trimmed to 75 bp to facilitate mapping using Bowtie v. 1.1.1 (performed in Tophat2 [32]). The trimmed reads were aligned to a reference HBV genome (isolate C_JPNAT, genotype C, accession #AB246345), and aligned reads were enumerated using Tophat2 with default parameters [32]. The coverage (% of maximum value) was plotted for the histogram. The maximum value for the Input was 25.97 reads per million (RPM), while that for the MeRIP sample (m6A) appeared to be 166.57 RPM, indicating successful enrichment.

#### 4.4.1. ELISA

HBs antigen in the medium was monitored by ELISA using an HBsAg ELISA kit (XpressBio, Frederick, MD, USA) according to the manufacturer’s instruction. Before ELISA assay, the medium was centrifuged and filtered. To avoid measurement at excessive concentration (which means possibility of saturation), serial dilutions were made, and the linearity was confirmed. 

#### 4.4.2. MTS Assay

Cell viability was examined by MTS assay, using a CellTiter 96 AQueous One Solution Cell Proliferation Assay kit (Promega, Madison, WI, USA) and Multiskan FC microplate reader (ThermoFisher Scientific), according to the manufacturer’s instructions.

#### 4.4.3. Knockdown by siRNA

siRNAs were transfected into HepG2/NTCP cells using Lipofectamine RNAiMAX reagent (ThermoFisher Scientific) 1 day before and 3 days after HBV infection (4 days after the initial siRNA transfection). The sequences of the siRNAs for the control and METTL3 were as follows: siControl, GCAGAGCUGGUUUAGUGAAtt (sense) and UUCACUAAACCAGCUCUGCtt (antisense); siMETTL3i, GCGAGGUCUCCUACAAGAUtt (sense) and AUCUUGUAGGAGACCUCGCtt (antisense); siMETTL3ii, GCAAGUAUGUUCACUAUGAtt (sense) and UCAUAGUGAACAUACUUGCtt (antisense).

#### 4.4.4. Western Blotting

Western blotting was performed as described previously [31]. Cells were washed with PBS and centrifuged at low speed. The precipitate was solubilized in the sample buffer containing sodium dodecyl sulfate (SDS) and 2-mercaptoethanol by sonication, which was followed by heat treatment. The samples were loaded and electrophoresed in the SDS-polyacrylamide gel, which was followed by electroblotting to the PVDF membrane. The membrane was labeled using indicated antibodies, which was followed by chemiluminescence. 

## Data Availability

All data will be available upon request.
